# XBP1 Regulates the Transcription of HIF-1a in BALB/c Mice with Chronic Rhinosinusitis without Polyps

**DOI:** 10.1155/2022/3066456

**Published:** 2022-07-23

**Authors:** Xiaopeng Qu, Hongyan Li, Lingzhao Meng

**Affiliations:** Department of Otolaryngology Head and Neck Surgery, Beijing Tiantan Hospital, Capital Medical University, Beijing 100070, China

## Abstract

X-box binding protein 1 (XBP1) is a transcription factor that recognizes the CRE-like element in enhancers of human T-cell leukemia virus and MHC class II gene and induces their transcription. This study was performed to characterize the function of XBP1, which was identified to be a differentially expressed gene via GEO database, in chronic rhinosinusitis (CRS) without nasal polyps (CRSsNP). XBP1 expression was significantly elevated in both CRSsNP patients and mice who were accompanied with mucosal thickening, goblet cell hyperplasia and chemosis, glandular hyperplasia, and dense infiltration of inflammatory cells. Silencing of XBP1 suppressed the development of CRSsNP in mice. Mechanistically, knockdown of XBP1 downregulated the expression of hypoxia-inducible factor 1-alpha (HIF-1a), and overexpression of XBP1 led to the opposite result. Silencing of HIF-1a inhibited *β*-catenin expression and impaired the Wnt/*β*-catenin pathway. Further overexpression of HIF-1a in XBP1-silenced CRSsNP mice exacerbated pathological changes in mouse nasal mucosal tissues, promoted inflammation, and activated the Wnt/*β*-catenin pathway. Taken together, overexpression of XBP1 may be associated with increased expression of HIF-1a and possibly contribute to the Wnt/*β*-catenin pathway activation and the development of CRSsNP.

## 1. Introduction

Chronic rhinosinusitis (CRS), a common disease disturbing more than 10% of the adult in Europe and the USA, is defined as persistent sinonasal symptoms lasting for at least 12 weeks along with sinonasal inflammation via computed tomography (CT) scan of paranasal sinuses or nasal endoscopy [1]. CRS is classified into CRS with (CRSwNP) and CRS without nasal polyps (CRSsNP) which represent about 20% and 80% of patients with CRS, respectively [2]. Both phenotypes are characterized by an overwhelming burden and an overlapping spectrum of symptoms, including facial pain or pressure, nasal discharge, congestion, and hyposmia or anosmia [3]. It has been suggested that CRSwNP is related to underlying T helper 2- (Th2-) driven inflammation (producing IFN-*γ*), while CRSsNP is precipitated by T helper 1- (Th1-) driven inflammation (secreting IL-4, IL-5, and IL-13) [4, 5]. However, type 2 immune responses were also observed in CRSsNP following similar patterns but less pronounced in CRSwNP [2]. Sinonasal epithelium composes a barrier between the outer and inner environment and is critical for protecting the mucosal interior from damage induced by inhaled pathogens, allergens, and other irritants [6]. Therefore, clarifying the underlying mechanisms regarding epithelial barrier dysfunction and inflammatory cell infiltration in CRSsNP is an essential work.

In the present study, we identified X-box binding protein 1 (XBP1) as a significantly overexpressed gene in nasal epithelial cells from CRSsNP patients relative to health controls in the Gene Expression Omnibus (GEO) database involving CRSsNP subset. The human XBP1 gene lies in chromosome 12 at position 22q12.1 consists of six exons within a 1.8 kb region which spans from 28,794,555 bp to 28,800,597 bp [7]. XBP1 translocates into the nucleus to enhance the transcription of genes to alleviate endoplasmic reticulum stress [8]. More recently, targeting XBP1 has been reported to inhibit the inflammatory response, thus offering a potential option to treat CRSwNP [9]. In addition, XBP1 has been proposed to act as a hub gene to regulate epithelial DNA damage responses [10]. However, the regulatory role of XBP1 in CRSsNP remains largely unclear. Since XBP1 mainly exerts its function as a transcription factor, we sought to decipher its possible downstream target. Hypoxia-induced factor-1*α* (HIF-1a) was revealed by hTFtarget website (http://bioinfo.life.hust.edu.cn/hTFtarget#!) as a candidate. Interestingly, HIF-1a has been demonstrated to play an imperative role in allergic rhinitis and CRS, highlighting its role as a major therapeutic target of these diseases in the future [11]. Thus, the current study is aimed at exploring whether or not XBP1 as a transcription factor might be involved in HIF-1a-mediated epithelial dysfunction and mucosal inflammation in the development of CRSsNP.

## 2. Materials and Methods

### 2.1. Patients and Tissue Samples

A total of 25 patients with CRSsNP and 30 patients with nasal septal deviation (without other nasal inflammatory diseases) admitted to Beijing Tiantan Hospital, Capital Medical University from June 2019 to December 2020 were recruited as study participants in this study. Diagnoses were made by nasal endoscopy and sinonasal CT scan. All patients were treated surgically. The sinus mucosa was collected from the CRSsNP patients, and inferior turbinate tissues and epithelial tissues from patients with nasal septal deviation were selected as controls. Patients with posterior nasal polyp, cystic fibrosis, fungal sinusitis, primary cilia, or gastroesophageal reflux were excluded. None of the study participants had taken oral glucocorticoids for at least 3 months prior to surgery, nor undertook aerosol corticosteroids within 1 month prior to surgery. Written consent forms were obtained from all participants before inclusion in the study. This research was permitted by the Institutional Ethics Committees of the Beijing Tiantan Hospital, Capital Medical University following the principles outlined by the *Declaration of Helsinki.*

### 2.2. Cell Culture

Human nasal epithelial cells (HNEPC) were purchased from iCell Bioscience Inc. (Shanghai, China). The cells were grown in an incubator at 37°C and 5% CO_2_ using airway epithelial cell growth medium (PromoCell, Heidelberg, Germany). The cells at logarithmic growth phase were transfected with oe-NC or oe-XBP1 (RiboBio Co., Ltd., Guangzhou, Guangdong, China). Transfection was performed using the Lipofectamine 2000 transfection kit (Thermo Fisher Scientific Inc., Waltham, MA, USA) according to the instructions. The transfected cells were added to airway epithelial cell growth medium and incubated in a 5% CO_2_, 37°C incubator until stable state for subsequent experiments.

### 2.3. Mouse Model Establishment

Thirty-six BALB/c mice (4-6 weeks, weighing 20.8 ± 2.6 g) were purchased from Beijing Vital River Laboratory Animal Technology Co., Ltd. (Beijing, China). All animals were included in accordance with the guidelines set forth in the Guide for the Care and Use of Laboratory Animals and approved by the Animal Care and Use Committees of Beijing Tiantan Hospital, Capital Medical University. The mice were housed in a specific-pathogen-free room and given adequate food and water. A 12-12 h light on-off cycle was maintained in an environmentally controlled room (25°C, 45% humidity).

The mice were randomly divided into 6 groups (*n* = 6) after 1 week of adaptation feeding: the control group (normal mice), CRSsNP (CRSsNP modeled mice), CRSsNP+sh-NC (CRSsNP modeled mice treated with lentivirus-encapsulated short hairpin RNA (sh-) negative control (NC)), CRSsNP+sh-XBP1 (CRSsNP modeled mice treated with lentivirus-encapsulated sh-XBP1), CRSsNP+sh-XBP1+oe-NC (CRSsNP modeled mice treated with lentivirus-encapsulated sh-XBP1 and oe-NC), and CRSsNP+sh-XBP1+oe-HIF-1a (CRSsNP modeled mice treated with lentivirus-encapsulated sh-XBP1 and oe-HIF-1a) groups. The lentiviruses used were purchased from VectorBuilder (Guangzhou, Guangdong, China), and the virus titer was 1 × 10^9^ TU/mL.

For the establishment of mice with CRSsNP, autoclaved hemostatic Merocel was firstly infiltrated with 20 *μ*L Gluconococcus aureus solution and subsequently inserted into the right nasal cavity of anesthetized mice, causing the blockage of the paranasal sinuses. Control mice were not treated. After the operation, the mice were bred normally for 90 d. After 90 d, the mice were euthanized through 150 mg/kg pentobarbital sodium and diagnosed as CRSsNP by pathological examination, and the nasal mucosal tissues were subsequently collected [12]. For lentivirus treatment, on the basis of the CRSsNP modeling, we administered 20 *μ*L lentiviruses to the mice intranasally on the 2^nd^ d after the operation [13], followed by the same steps as for the CRSsNP model establishment.

### 2.4. Periodic Acid-Schiff (PAS) Staining

PAS staining kit (Beijing Solarbio Life Sciences Co., Ltd., Beijing, China) was used to detect mucin expression in epithelial cells of nasal mucosal tissues. The sections were dewaxed and hydrated with water, then oxidized in 0.5% periodate solution, and rinsed in distilled water. The sections were stained with Coleman's feulgen solution for 15 min, rinsed with running tap water for 10 min, and counter-stained with hematoxylin for 1 min. After washing for 5 min, the sections were differentiated in 1% acidic alcohol for 30 s, treated with 0.2% ammonia or saturated lithium carbonate solution for 1 min, routinely dehydrated, cleared, and sealed. Images were acquired using an inverted microscope (Nikon, Tokyo, Japan), and the percentage of PAS-positive cells was evaluated [14].

### 2.5. Hematoxylin-Eosin (HE) Staining

HE staining of nasal mucosal tissues was performed in strict accordance with the instructions of the HE staining kit (Beyotime, Shanghai, China) to examine eosinophil infiltration. The nasal mucosal tissues were embedded, sectioned, dewaxed with xylene for 8 min, soaked in gradient concentration alcohol and then immersed in water for 5 min, and moved into hematoxylin for 15 min. After that, the sections were treated with 1% hydrochloric acid alcohol for 30 s for differentiation. The sections were stained with eosin solution for 5 min, dehydrated in gradient concentration alcohol, cleared with xylene for 10 min, and sealed with gum (Millipore Corp, Billerica, MA, USA). The completed tissue sections were observed under a light microscope (Eclipse TE2000-U, Nikon), and five fields of view were randomly selected for photography. The degree of submucosal inflammatory cell infiltration was scored on the following scale: 0: none, 1: mild occasional scattered inflammatory cells, 2: moderate inflammatory infiltration with partial inflammatory infiltration, and 3: severe diffuse infiltration of inflammatory cells [15].

### 2.6. Enzyme-Linked Immunosorbent Assay (ELISA)

The concentrations of inflammatory factors IFN-*γ*, IL-1*β*, and IL-6 in nasal mucosal tissues were assessed using ELISA assays. A total of 1 mL 0.9% NaCl was added to each 0.1 g sample, and all samples were homogenized at 1000 rpm for 5 min and centrifuged at 1500 × g for 10 min at 4°C to collect the supernatant. Concentrations of inflammatory factors IFN-*γ* (DIF50C, R&D Systems, Minneapolis, MN, USA), IL-1*β* (DLB50, R&D Systems), IL-6 (D6050, R&D Systems), IL-4 (PI618, Beyotime), and IL-13 (ab47353, Abcam, Cambridge, MA, USA) in nasal mucosal tissues of patients with CRSsNP and controls were measured using ELISA kits.

The concentrations of inflammatory factors IFN-*γ* (MIF00, R&D Systems), IL-1*β* (MLB00C, R&D Systems), IL-6 (M6000B, R&D Systems), IL-4 (PI612, Beyotime), and IL-13 (ab219634, Abcam) in nasal mucosal tissues of mice in the CRSsNP and control groups were assessed using ELISA kits as well. All operations were performed strictly in accordance with the ELISA kit instructions.

### 2.7. RNA Extraction, Reverse Transcription, and PCR

Total RNA was extracted from nasal mucosal tissues using TRIzol (Invitrogen, Carlsbad, CA, USA) as per the manufacturer's instructions. The concentration and quality of total RNA were assessed using a Nanodrop spectrophotometer (Thermo Fisher Scientific). Reverse transcription was performed where cDNA was synthesized using the PrimeScript™ RT reagent kit (Takara, Tokyo, Japan), and all operations were performed according to the instructions for the kit. We performed real-time quantitative polymerase chain reaction (qPCR) on a CFX96 Real-Time PCR Detection System (Bio-Rad Laboratories, Hercules, CA, USA) using TB Green® Premix Ex Taq™ (Takara). The housekeeping gene was glyceraldehyde-3-phosphate dehydrogenase (GAPDH), and the relative gene expression was calculated using the comparative 2^-*ΔΔ*Ct^ method. Gene primer sequences used are shown in Table 1.

### 2.8. Western Blot

Total protein was extracted by adding radioimmunoprecipitation assay lysis buffer (Solarbio) to mouse nasal mucosa tissues, and protein concentration was measured using a bicinchoninic acid kit (Sigma). Samples containing 20 *μ*g of protein were boiled, and the proteins were separated by 10% polyacrylamide gel electrophoresis. The separated proteins were transferred to polyvinylidene fluoride membranes (Millipore). The membrane was sealed in 5% skim milk (BD, San Jose, CA, USA) for 1 h at room temperature and then incubated with primary antibodies to XBP1 (1 : 1000, ab220783, Abcam), HIF-1a (1 : 2000, GTX127309, GeneTex, Inc., Alton Pkwy Irvine, CA, USA), HIF-2a (1 : 1000, ab109616, Abcam), HIF-3a (1 : 1000, GTX85702, GeneTex), *β*-catenin (1 : 1000, ab223075, Abcam), and GAPDH (1 : 10,000, ab181603, Abcam) overnight at 4°C. The membranes were then incubated with goat anti-rabbit secondary antibody (1 : 5000, ab205718, Abcam) for 60 min at 37°C and developed by exposure to an enhanced chemiluminescence substrate kit (Abcam). Gray value analysis of the target gene was performed by Image J (National Institutes of Health, Bethesda, MD, USA) with GAPDH as the internal reference gene.

### 2.9. Chromatin Immunoprecipitation (ChIP) Assay

ChIP analysis was performed using the EZ-Magna ChIP™ A/G ChIP Kit (17-10086, Sigma-Aldrich). After being fixed for 10 min at room temperature using 4% paraformaldehyde, the cells were sonicated and centrifuged at 12,000 × g for 10 min at 4°C to remove insoluble precipitates. Subsequently, the samples were cultured with protein G agarose beads at 4°C for 1 h and centrifuged at 5000 × g for 1 min at 4°C to collect the supernatant. The supernatant was hybridized with the antibodies against XBP1 (1 : 50, ab220783, Abcam) or rabbit anti-IgG isotype control (1 : 100, ab172730, Abcam) overnight at 4°C. The precipitated protein-DNA complex was incubated with protein G agarose beads at 4°C for 60 min. After centrifugation at 5000 × g for 1 min, the supernatant was discarded. The protein-DNA complexes were fragmented overnight at 65°C. The DNA fragments were recovered and used as amplification template for qPCR.

### 2.10. Dual-Luciferase Reporter Gene Assay

Potential binding sites of the HIF-1a promoter to XBP1 were obtained from JASPAR (http://jaspar.genereg.net/). The binding site of XBP1 on the HIF-1a promoter region was cloned into the firefly luciferase reporter vector pGL3 (Promega, Madison, WI, USA) to construct a luciferase reporter plasmid for Promoter. The above plasmids were cotransfected into HNEPC with oe-NC or oe-XBP1, respectively, according to the manufacturer's instructions. Forty-eight hours after transfection, relative luciferase activity was assessed using a dual-luciferase reporter system (Promega).

### 2.11. Statistics

SPSS statistical software version 22.0 (IBM, Chicago, IL, USA) was used to analyze the data. For the *in vitro* assays, data were expressed as the mean ± standard deviation from 3 independent experiments. An unpaired *t* test was applied to compare the unpaired data between two groups. Data among multiple groups were compared using one-way or two-way analysis of variance (ANOVA), with a Tukey's test conducted for a post hoc test. In all cases, differences were considered statistically significant at *p* < 0.05.

## 3. Results

### 3.1. XBP1 Is Highly Expressed in CRSsNP Patients and CRSsNP Mice

**Figure 1:**
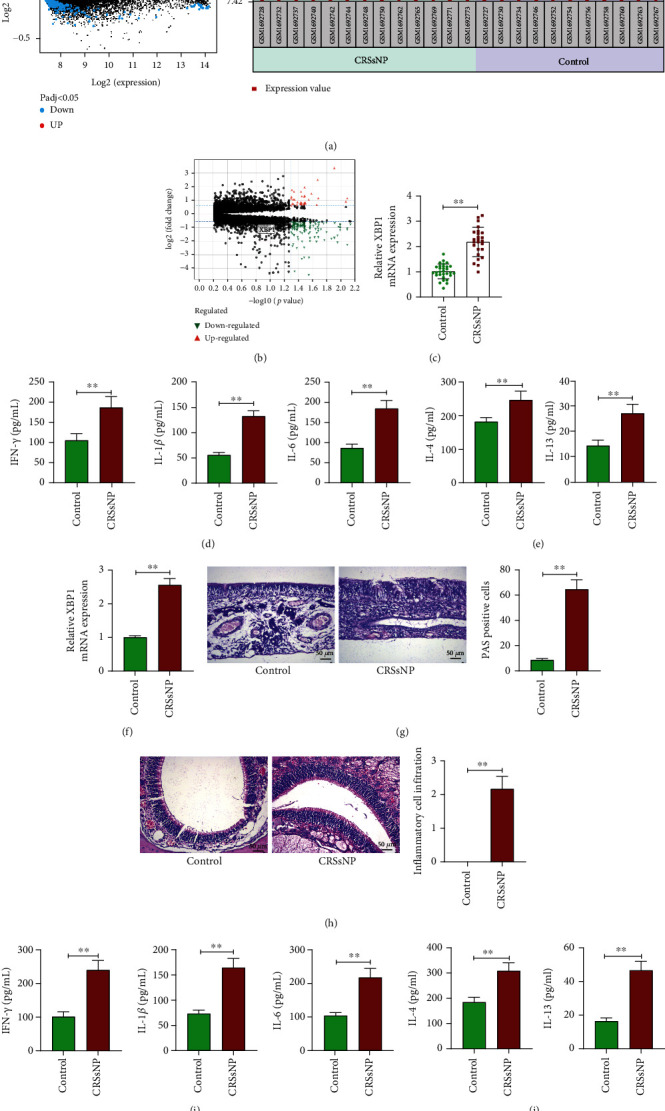
XBP1 is highly expressed in CRSsNP patients and CRSsNP mice. (a) Highly expressed XBP1 in CRSsNP screened using the GSE69093 dataset. (b) Analysis of differentially expressed genes of in the GSE194282 dataset. (c) RT-qPCR analysis of XBP1 expression in collected nasal mucosal tissue samples from CRSsNP patients and controls. (d) The levels of inflammatory factors IFN-*γ*, IL-1*β*, and IL-6 in nasal mucosal tissues of patients with CRSsNP and controls measured using ELISA. (e) The levels of inflammatory factors IL-4 and IL-13 in nasal mucosal tissues of patients with CRSsNP and controls measured using ELISA. (f) RT-qPCR analysis of XBP1 expression in nasal mucosal tissue samples from CRSsNP mice and controls. (g) Mucin expression in nasal mucosal epithelial cells of CRSsNP mice and controls examined using PAS staining. (h) Pathological changes of nasal mucosa in mice with CRSsNP and controls observed by HE staining. (i) The levels of inflammatory factors IFN-*γ*, IL-1*β*, and IL-6 in nasal mucosal tissues of mice with CRSsNP and controls measured using ELISA. (j) The levels of inflammatory factors IL-4 and IL-13 in nasal mucosal tissues of mice with CRSsNP and controls measured using ELISA. ^∗∗^*p* < 0.01 versus the control participants or mice. The data are measurement data, presented as mean ± SD. Data between two groups were compared using an unpaired *t* test. The experiment was independently repeated three times. *n* = 6.

We selected nasal mucosal epithelial cell samples from CRSsNP patients (GSM1692728, GSM1692732, GSM1692737, GSM1692740, GSM1692742, GSM1692744, GSM1692748, GSM1692750, GSM1692762, GSM1692765, GSM1692769, GSM1692771, and GSM1692773) and from healthy controls (GSM1692727, GSM1692730, GSM1692734, GSM1692746, GSM1692752, GSM1692754, GSM1692756, GSM1692758, GSM1692760, GSM1692763, and GSM1692767) from the GEO database GSE69093 dataset to screen for differentially expressed genes in CRSsNP. Significant overexpression of XBP1 (GPL10558 platform number: ILMN_1809433) was identified (Figure 1(a)). While in another CRSwNP-related dataset GSE194282, we found that XBP1 was not differentially expressed in CRSwNP tissues relative to control uncinate tissues (*n* = 7) (Figure 1(b)).

Our RT-qPCR analysis of nasal mucosal tissue samples collected from CRSsNP patients and controls (without other inflammatory nasal diseases) revealed that XBP1 was highly expressed in the nasal mucosal tissues of CRSsNP patients (Figure 1(c)). The levels of type 1 inflammatory factors IFN-*γ*, IL-1*β*, and IL-6 and T2 inflammatory factors IL-4 and IL-13 were found to be significantly enhanced in the nasal mucosal tissues of patients with CRSsNP by ELISA (Figures 1(d) and 1(e)).

We induced a mouse model of CRSsNP (Supplementary Figure S1), and the expression of XBP1 was significantly overexpressed in the nasal mucosal tissues of CRSsNP mice compared with the control mice (Figure 1(f)). The positivity of mucin in nasal mucosal epithelial cells was upregulated (Figure 1(g)), which was occurred concomitant with the thickened nasal mucosal tissues, significant hyperplasia of goblet cells and glands, and a significant increase in inflammatory cell infiltration (Figure 1(h)). Consistently, the results of ELISA showed that the levels of type 1 inflammatory factors IFN-*γ*, IL-1*β*, and IL-6 in nasal mucosal tissues were significantly augmented (Figure 1(i)). Levels of type 2 inflammatory factors IL-4 and IL-13 were also significantly increased (Figure 1(j)).

### 3.2. Downregulation of XBP1 Inhibits the Development of CRSsNP in Mice

**Figure 2:**
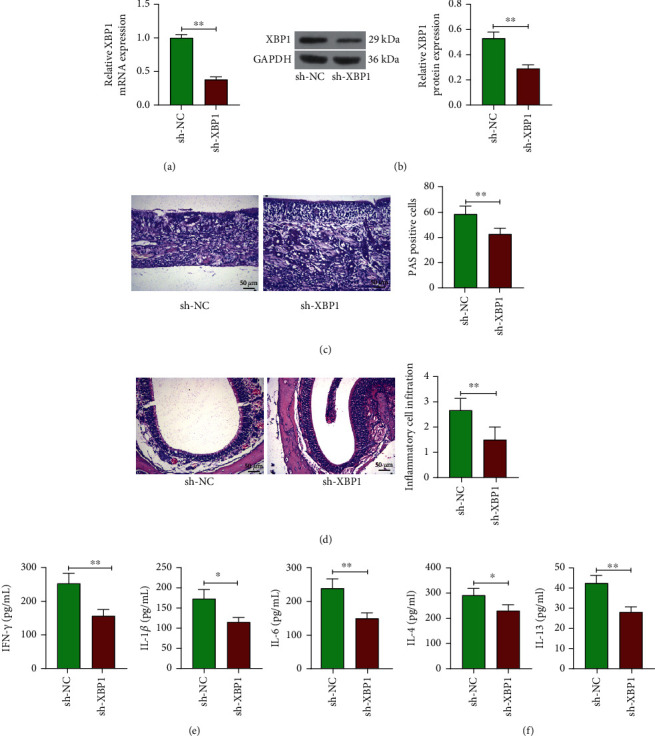
XBP1 knockdown alleviates the development of CRSsNP in mice. (a, b) Detection of mRNA and protein expression of XBP1 in nasal mucosa tissues of mice after downregulation of XBP1 by (a) RT-qPCR and (b) Western blot. (c) PAS staining analysis of mucin expression in nasal mucosal epithelial cells of mice. (d) Pathological changes of nasal mucosa in mice observed by HE staining. (e) The levels of inflammatory factors IFN-*γ*, IL-1*β*, and IL-6 in nasal mucosal tissues of mice measured using ELISA. (f) The levels of inflammatory factors IL-4 and IL-13 in nasal mucosal tissues of mice measured using ELISA. ^∗^*p* < 0.05 and ^∗∗^*p* < 0.01 versus the CRSsNP mice administrated with sh-NC. The data are measurement data, presented as mean ± SD. Data between two groups were compared using an unpaired *t* test. The experiment was independently repeated three times. *n* = 6.

To investigate the specific role of XBP1 in CRSsNP, we inhibited the expression of XBP1 in CRSsNP mice by intranasal instillation of lentivirus-encapsulated sh-XBP1. The expression of XBP1 in nasal mucosal tissues of mice was detected by RT-qPCR and Western blot. The mRNA and protein expression of XBP1 in nasal mucosal tissues was found to be significantly decreased after intranasal instillation of sh-XBP1 (Figures 2(a) and 2(b)). PAS staining analysis demonstrated a significant decrease in the cellular positivity of mucin in nasal mucosal epithelial cells after inhibition of XBP1 expression (Figure 2(c)). Meanwhile, the pathological changes of mouse nasal mucosal tissues were detected by HE staining. The results showed that the nasal mucosa was thinned, goblet cell and glandular hyperplasia were significantly reduced, and inflammatory cell infiltration was significantly decreased after XBP1 downregulation (Figure 2(d)). The ELISA assay demonstrated that the levels of IFN-*γ*, IL-1*β*, IL-6, IL-4, and IL-13 were significantly reduced in the nasal mucosal tissues of mice after silencing of XBP1 (Figures 2(e) and 2(f)). Our findings suggest that silencing of XBP1 in CRSsNP mice attenuates pathological changes in mouse nasal mucosal tissues, suppresses inflammation, and hinders the development of CRSsNP.

### 3.3. XBP1 Promotes the Expression of HIF-1a

**Figure 3:**
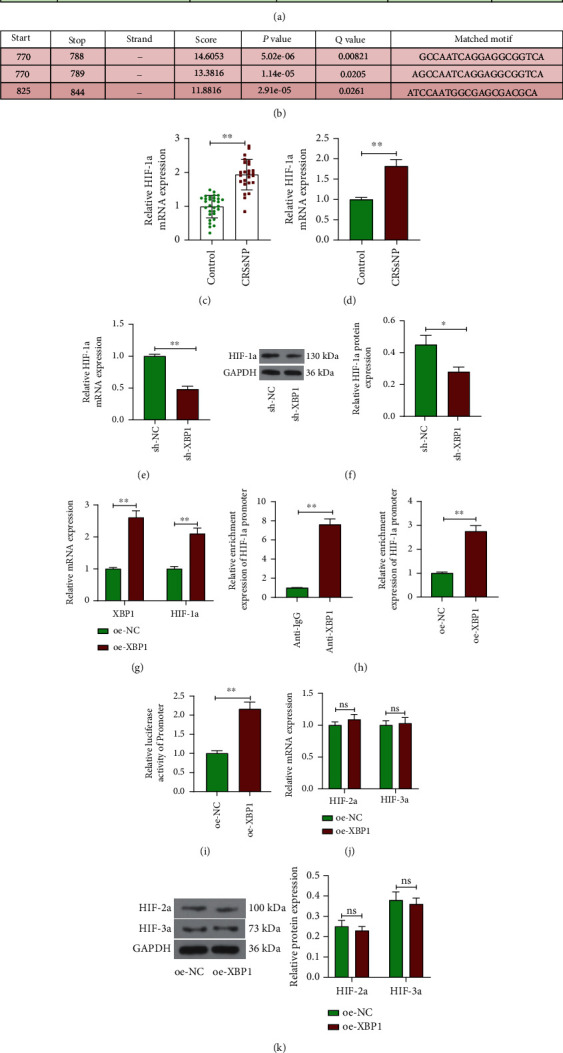
XBP1 promotes the expression of HIF-1a. (a) HIF-1a was predicted as a downstream gene of XBP1 in the hTFtarget database. (b) Potential binding sites of the HIF-1a promoter to XBP1. (c) RT-qPCR analysis of HIF-1a expression in nasal mucosal tissues from CRSsNP patients and controls. (d) RT-qPCR analysis of HIF-1a expression in nasal mucosal tissues from CRSsNP mice and controls. (e) RT-qPCR and (f) Western blot analyses of XBP1 mRNA and protein expression in mice. (g) Detection of mRNA expression of XBP1 and HIF-1a in cells by RT-qPCR. (h) The enrichment ability of anti-XBP1 on HIF-1a promoter assessed using ChIP-qPCR. (i) The effect of overexpression of XBP1 on promoter luciferase activity of HIF-1a measured using dual-luciferase assay. (j) The effect of overexpression of XBP1 on HIF-2a and HIF-3a mRNA expression by RT-qPCR assay. (k) The effect of overexpression of XBP1 on HIF-2a and HIF-3a protein expression by Western blot assay. ^∗^*p* < 0.05 and ^∗∗^*p* < 0.01 versus the CRSsNP mice administrated with sh-NC or HNEPC transfected with oe-NC. The data are measurement data, presented as mean ± SD. Data between two groups were compared using an unpaired *t* test. Data among multiple groups were compared using two-way analysis of variance and then analyzed with Tukey's post hoc test. The experiment was independently repeated three times. *n* = 6.

To investigate the mechanism of XBP1 action in CRSsNP, we analyzed the downstream genes of XBP1. Through the hTFtarget database (http://bioinfo.life.hust.edu.cn/hTFtarget#!), XBP1 was predicted to bind to the promoter of HIF-1a (Figure 3(a)).  Figure 3(b) shows the specific binding sites between XBP1 and the HIF-1*α* promoter. HIF-1a was found to be significantly highly expressed in CRSsNP patients and mouse nasal mucosal tissue samples by RT-qPCR analysis (Figures 3(c) and 3(d)). The expression of HIF-1a was significantly reduced in the nasal mucosal tissues of CRSsNP mice with sh-XBP1 (Figures 3(e) and 3(f)). Meanwhile, the expression of both XBP1 and HIF-1a was significantly increased after transfection of oe-XBP1 in stably growing HNEPC (Figure 3(g)). Then, ChIP-qPCR and dual-luciferase reporter assay were used to explore the relationship between XBP1 and HIF-1a. Overexpression of XBP1 significantly increased the enrichment of anti-XBP1 in the HIF-1a promoter region (Figure 3(h)) and the luciferase activity of the promoter luciferase vector (Figure 3(i)). In addition, we performed RT-qPCR and Western blot assay on HIF-1-associated proteins HIF-2a and HIF-3a after overexpression of XBP1 and found that overexpression of XBP1 had no effect on their expression (Figures 3(j) and 3(k)). These results showed that XBP1 could specifically bind to HIF-1a and promote its expression.

### 3.4. Silencing of XBP1 Inhibits the Wnt/*β*-Catenin Pathway in CRSsNP

**Figure 4:**
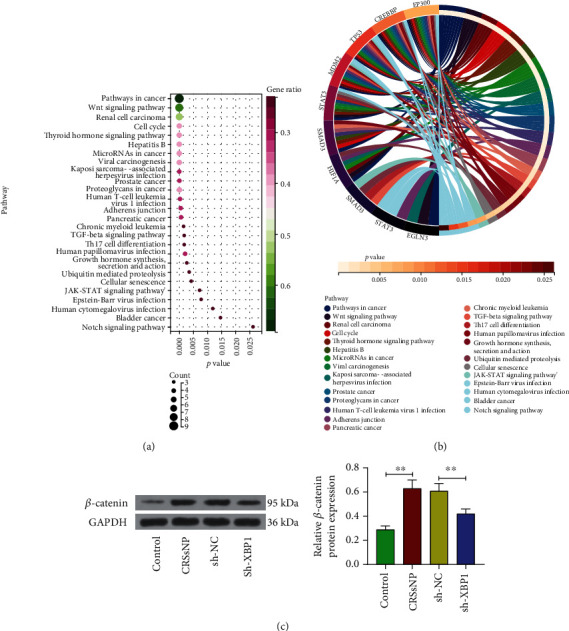
Silencing of HIF-1a inhibits the Wnt/*β*-catenin pathway. (a) KEGG enrichment analysis of genes associated with HIF-1a and (b) visualization by bubble mapping. (c) Western blot detection of protein expression of *β*-catenin in nasal mucosal tissues of CRSsNP mice. ^∗∗^*p* < 0.01 versus the control mice or CRSsNP mice administrated with sh-NC. The data are measurement data, presented as mean ± SD. Data among multiple groups were compared using one-way analysis of variance and then analyzed with Tukey's post hoc test. The experiment was independently repeated three times. *n* = 6.

To further determine the mechanism of HIF-1a action in CRSsNP, we analyzed HIF-1a-related genes by KEGG enrichment and visualized them by bubble mapping. It was found that HIF-1a-related genes could be enriched in the Wnt signaling pathway (Figures 4(a) and 4(b)). Western blot results exhibited that the expression of *β*-catenin protein was elevated in the nasal mucosal tissues of CRSsNP mice, which was significantly downregulated in CRSsNP mice with silenced XBP1 expression (Figure 4(c)).

### 3.5. HIF-1a Reverses the Attenuating Effect of Sh-XBP1 on the Development of CRSsNP in Mice

**Figure 5:**
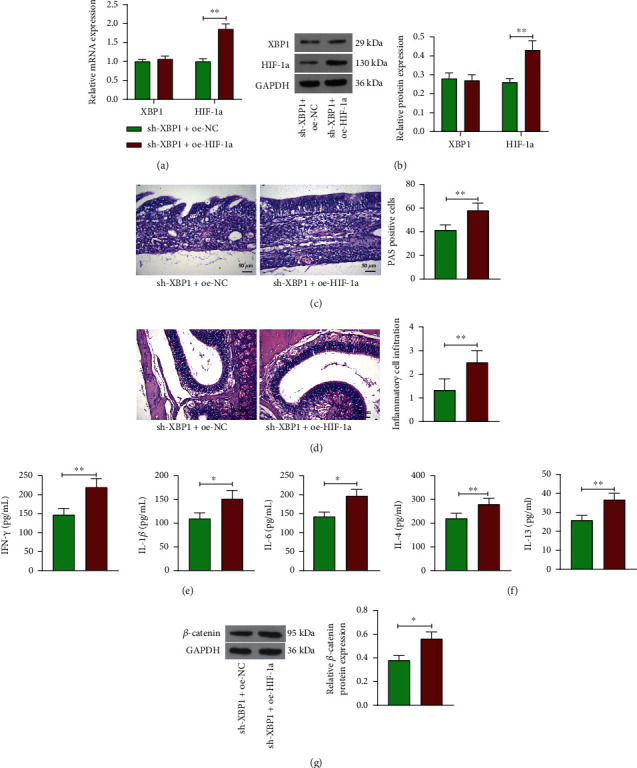
Overexpression of HIF-1a reverses the alleviating effect of sh-XBP1 on the development of CRSsNP in mice. (a, b) Analysis of mRNA and protein expression of XBP1 and HIF-1a after further overexpression of HIF-1a in CRSsNP mice with silenced XBP1 expression by (a) RT-qPCR and (b) Western blot. (c) PAS staining analysis of mucin expression in nasal mucosal epithelial cells of mice. (d) Pathological changes of nasal mucosa in mice observed by HE staining. (e) The levels of inflammatory factors IFN-*γ*, IL-1*β*, and IL-6 in nasal mucosal tissues of mice measured using ELISA. (f) The levels of inflammatory factors IL-4 and IL-13 in nasal mucosal tissues of mice measured using ELISA. (g) Western blot detection of protein expression of *β*-catenin in nasal mucosal tissues of CRSsNP mice. ^∗^*p* < 0.05 and ^∗∗^*p* < 0.01 versus the CRSsNP mice administrated with sh-XBP1+oe-NC. The data are measurement data, presented as mean ± SD. Data between two groups were compared using an unpaired *t* test. Data among multiple groups were compared using one-way analysis of variance and then analyzed with Tukey's post hoc test. The experiment was independently repeated three times. *n* = 6.

To assess the role of the XBP1/HIF-1a axis in CRSsNP, we further overexpressed HIF-1a in CRSsNP mice with sh-XBP1. After coadministration, the expression of HIF-1a was significantly elevated, and the expression of XBP1 was not affected (Figures 5(a) and 5(b)). PAS staining analysis showed that further upregulation of HIF-1a significantly increased the cellular positivity of mucin in mouse nasal mucosal epithelial cells (Figure 5(c)). Also, after further overexpression of HIF-1a, the nasal mucosa was thickened, with significantly enhanced goblet cells and glandular hyperplasia and increased inflammatory cell infiltration (Figure 5(d)). The levels of IFN-*γ*, IL-1*β*, and IL-6 were significantly upregulated in mouse nasal mucosal tissues after further overexpression of HIF-1a in the presence of sh-XBP1 (Figure 5(e)). Meanwhile, the levels of type 2 inflammatory factors IL-4 and IL-13 were elevated as well following the overexpression of HIF-1a (Figure 5(f)). Western blot analysis showed that upregulation of HIF-1a promoted *β*-catenin protein expression and activated the Wnt/*β*-catenin pathway (Figure 5(g)). The above results suggest that further overexpression of HIF-1a partially reverses the hindering effect of sh-XBP1 on the development of CRSsNP in mice by exacerbating pathological changes in mouse nasal mucosal tissues, promoting inflammation, and activating the Wnt/*β*-catenin pathway.

## 4. Discussion

It has been recently reported that CRSsNP has at least three main inflammatory endotypes (T1, T2, and T3) that are governed by different molecular mechanisms and inflammatory cells [16]. Acute viral infections of the upper aerodigestive tract induce various proinflammatory cytokines, including IFN-*γ*, IL-1*β*, and IL-6 [17]. In this study, we have for the first time demonstrated that the transcription factor XBP1 might play an imperative role in the nasal mucosal tissues of CRSsNP. The expression of XBP1 was significantly boosted in CRSsNP subjects and mice. Furthermore, depletion of XBP1 alleviated pathological changes of nasal mucosa and inflammatory biomarkers (both types 1 and 2) by repressing the HIF-1a expression and the Wnt/*β*-catenin pathway activation, indicating a possible underlying role in the pathogenesis of CRSsNP.

Vidal et al. have long been established that XBP1 deficiency protected against the pathogenesis of Huntington's disease, an autosomal dominant neurodegenerative disease, in the YAC128 mouse model [18]. More relevantly, XBP1 expression was increased by neutrophil elastase stimulation in an immortalized human bronchial epithelial cell line 16HBE14o- and was involved in neutrophil elastase-induced MUC5AC expression [19]. In the current study, we observed elevated expression of XBP1 in both nasal mucosal tissue samples from CRSsNP patients and mice. Mucus hypersecretion in respiratory tract diseases results from several complex processes, including hyperplasia of mucin-secreting goblet cells in the epithelium and/or hyperplasia of submucosal glands [20]. In the study, depletion of XBP1 by intranasal instillations of lentivirus-encapsulated shRNAs successfully alleviated pathological changes of nasal mucosa, including hyperplasia of goblet cells and submucosal glands and reduced the levels of IFN-*γ*, IL-1*β*, IL-6, IL-4, and IL-13. In line with our results, silencing of XBP1 was capable of reversing the aggravating effect of SIRT1 knockout on hepatic ischemia-reperfusion injury in mice by decreasing IL-1*β*, TNF-*α*, and IL-18 [21]. Interestingly, XBP1 is an arm of the unfolded protein response that activates the hexosamine biosynthetic pathway, a pathway that mediates protein N-glycosylation and survival from ER stress-induced apoptosis in epithelial-mesenchymal transition [22]. Moreover, partial epithelial-mesenchymal transition occurred in patients with CRS, notably in CRSsNP patients [23]. Therefore, further studies are needed to clarify the role of XBP1 in regulating epithelial-mesenchymal transition in CRSsNP.

Subsequently, we examined the downstream target of XBP1.The levels of HIF-1a in relatively low eosinophilic infiltration tissue samples were higher than those in control and eosinophilic CRSwNP samples [24], and HIF-1a is related to neutrophilic inflammation and glucocorticoid resistance in CRSwNP patients [25]. Moreover, Khalil et al. evidenced that whole tissue from CRSwNP subjects showed promoted HIF-1a gene expression [26]. Previous evidence suggested that XBP1 promoted triple-negative breast cancer by controlling the HIF-1a pathway [27]. However, the binding relation between XBP1 and HIF-1a in CRSsNP, to the best of our knowledge, has not been reported before, which has been substantiated in HNEPC in the present study using ChIP-qPCR and dual-luciferase reporter assays. Our subsequent KEGG analysis showed that the Wnt/*β*-catenin signaling pathway was enriched by downstream genes of HIF-1a. Böscke et al. revealed that Wnt/*β*-catenin signaling sustains mucosal inflammation and contributes to a vast network of change during epithelial remodeling in CRSwNP [28]. Still, its function in CRSsNP remains untouched and unanswered. Even though its regulatory role in CRSsNP is out of scope for this article, we managed to find that the protein expression of *β*-catenin was impaired after knockdown of XBP1 *in vivo*, which at least partially explained the possible linkage between the two in CRSsNP.

As we explained above, mucus secretion is of great importance for the maintenance of airway health, among which HIFs are believed to be involved in the modulation of mucin synthesis and regulation [29]. Here we found that overexpression of HIF-1a was able to overturn the effects of sh-XBP1 and enhanced the expression of mucus, the concentration of type 1 and type 2 inflammatory factors, and the protein expression of *β*-catenin. Consistently, HIF-1a deficiency in dendritic cells attenuated symptoms and inflammatory indicators in allergic rhinitis mice induced with OVA [30]. Similarly, Alexander et al. showed that deletion of HIF-1a in myeloid cells in OVA-induced asthma also reduced eosinophil infiltration and goblet cell hyperplasia [31]. The interactions between *β*-catenin, HIF-1a, and XBP1 have been well described under the context of hypoxia [32].

## 5. Conclusion

In conclusion, our finding that XBP1 was related to epithelial barrier dysfunction and inflammation in CRSsNP mice suggests that XBP1 might play an important role in the development of CRSsNP. Moreover, XBP1 might play its role in CRSsNP via HIF-1a-mediated regulation of the Wnt/*β*-catenin signaling pathway. A more comprehensive investigation about the effects of XBP1 on other immune cells should be conducted in the future. Besides, analyses of XBP1 expression in CRSwNP samples are needed to further support our statement.

## Figures and Tables

**Table 1:** Primer sequences used for PCR.

Name of primer	Sequences (5′-3′)
hsa-XBP1-F	CTGCCAGAGATCGAAAGAAGGC
hsa-XBP1-R	CTCCTGGTTCTCAACTACAAGGC
mmu-XBP1-F	TGGACTCTGACACTGTTGCCTC
mmu-XBP1-R	TAGACCTCTGGGAGTTCCTCCA
hsa-HIF-1a-F	TATGAGCCAGAAGAACTTTTAGGC
hsa-HIF-1a-R	CACCTCTTTTGGCAAGCATCCTG
mmu-HIF-1a-F	CCTGCACTGAATCAAGAGGTTGC
mmu-HIF-1a-R	CCATCAGAAGGACTTGCTGGCT
hsa-GAPDH-F	GTCTCCTCTGACTTCAACAGCG
hsa-GAPDH-R	ACCACCCTGTTGCTGTAGCCAA
mmu-GAPDH-F	CATCACTGCCACCCAGAAGACTG
mmu-GAPDH-R	ATGCCAGTGAGCTTCCCGTTCAG

Note: hsa: homo sapiens; mmu: mus musculus; XBP1: X-box binding protein 1; HIF-1a: hypoxia-inducible factor 1-alpha; GAPDH: glyceraldehyde-3-phosphate dehydrogenase; F: forward; R; reverse.

## Data Availability

All the data generated or analyzed during this study are included in this published article.

## Supplementary Materials

Supplementary Materials Supplementary Figure S1: a flowchart of animal treatment schedule.
