# Supplementary figures and images for: XBP1 Regulates the Transcription of HIF-1a in BALB/c Mice with Chronic Rhinosinusitis without Polyps
Source: Anal Cell Pathol (Amst). 2022 Jul 23;2022:3066456. doi: 10.1155/2022/3066456 (PMC9338878; doi:10.1155/2022/3066456)

**Supplementary Figure S1** A flowchart of animal treatment schedule.


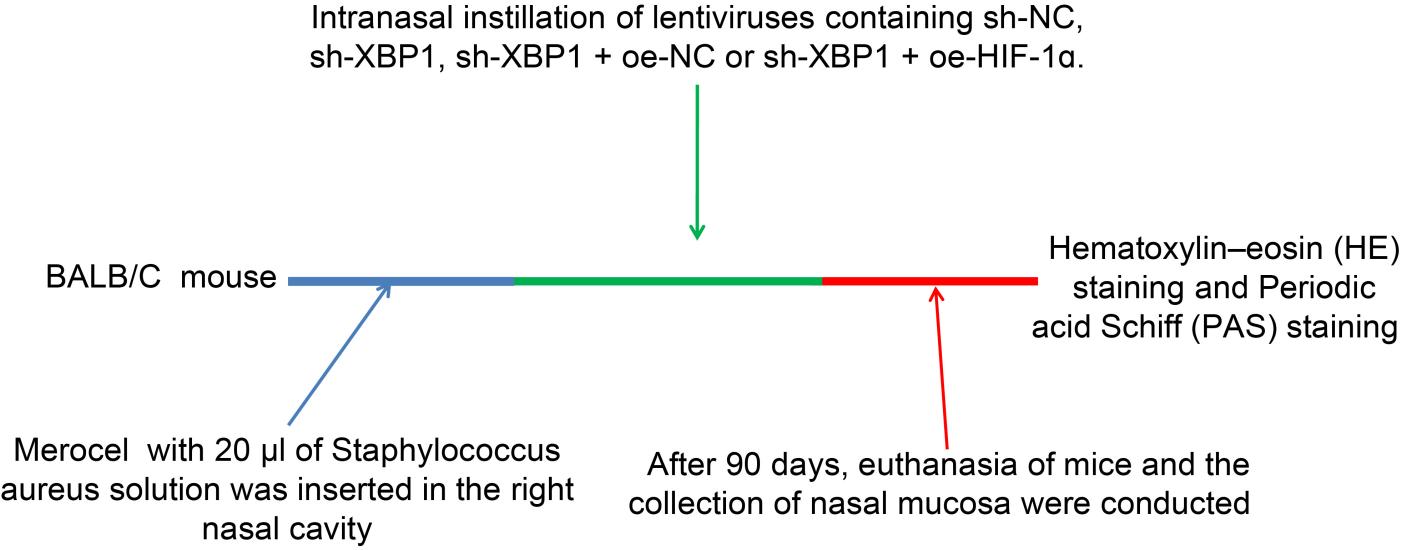

Supplement: Supplementary Materials — Supplementary Figure S1: a flowchart of animal treatment schedule. [file 3066456.f1.docx]
